# Estimation, Evaluation and Characterization of Carbapenem Resistance Burden from a Tertiary Care Hospital, Pakistan

**DOI:** 10.3390/antibiotics12030525

**Published:** 2023-03-06

**Authors:** Aamir Jamal Gondal, Nakhshab Choudhry, Hina Bukhari, Zainab Rizvi, Shah Jahan, Nighat Yasmin

**Affiliations:** 1 Department of Biomedical Sciences, King Edward Medical University, Lahore 54000, Pakistan; 2 Department of Biochemistry, King Edward Medical University, Lahore 54000, Pakistan; 3 Department of Pathology, King Edward Medical University, Lahore 54000, Pakistan; 4 Department of Oral Pathology, de’Montmorency College of Dentistry, Lahore 54000, Pakistan; 5 Department of Immunology, University of Health Sciences, Lahore 54600, Pakistan

**Keywords:** carbapenem resistance, carbapenemases, *Enterobacterales*, sequence types, Pakistan

## Abstract

Carbapenem resistance has become major concern in healthcare settings globally; therefore, its monitoring is crucial for intervention efforts to halt resistance spread. During May 2019–April 2022, 2170 clinical strains were characterized for antimicrobial susceptibility, resistance genes, replicon and sequence types. Overall, 42.1% isolates were carbapenem-resistant, and significantly associated with *Klebsiella pneumoniae* (*K. pneumoniae*) (*p* = 0.008) and *Proteus* species (*p* = 0.043). Carbapenemases were detected in 82.2% of isolates, with *bla*_NDM-1_ (41.1%) associated with the ICU (*p* < 0.001), cardiology (*p* = 0.042), pediatric medicine (*p* = 0.013) and wound samples (*p* = 0.041); *bla*_OXA-48_ (32.6%) was associated with the ICU (*p* < 0.001), cardiology (*p* = 0.008), pediatric medicine (*p* < 0.001), general surgery (*p* = 0.001), general medicine (*p* = 0.005) and nephrology (*p* = 0.020); *bla*_KPC-2_ (5.5%) was associated with general surgery (*p* = 0.029); *bla*_NDM-1_/*bla*_OXA-48_ (11.4%) was associated with general surgery (*p* < 0.001), and wound (*p* = 0.002), urine (*p* = 0.003) and blood (*p* = 0.012) samples; *bla*_OXA-48_/*bla*_VIM_ (3.1%) was associated with nephrology (*p* < 0.001) and urine samples (*p* < 0.001). Other detected carbapenemases were *bla*_VIM_ (3.0%), *bla*_IMP_ (2.7%), *bla*_OXA-48_/*bla*_IMP_ (0.1%) and *bla*_VIM_/*bla*_IMP_ (0.3%). Sequence type (ST)147 (39.7%) represented the most common sequence type identified among *K. pneumoniae*, along with ST11 (23.0%), ST14 (15.4%), ST258 (10.9%) and ST340 (9.6%) while ST405 comprised 34.5% of *Escherichia coli* (*E. coli*) isolates followed by ST131 (21.2%), ST101 (19.7%), ST10 (16.0%) and ST69 (7.4%). Plasmid replicon types IncFII, IncA/C, IncN, IncL/M, IncFIIA and IncFIIK were observed. This is first report describing the carbapenem-resistance burden and emergence of *bla*_KPC-2_-ST147, *bla*_NDM-1_-ST340 and *bla*_NDM-1_-ST14 in *K. pneumoniae* isolates and *bla*_NDM-1_-ST69 and *bla*_NDM-1_/*bla*_OXA-48_-ST69 in *E. coli* isolates coharboring extended-spectrum beta-lactamases (ESBLs) from Pakistan.

## 1. Introduction

Since the glorious discovery of first antibiotic, revolutionary changes occurred in the health care settings that helped in reducing the suffering of mankind by preventing the onset of infectious diseases [1]. However, due to misuse of antibiotics, the mounting rise in antimicrobial resistance (AMR) has posed greater clinical challenges and public health threats with every passing day as accepted by health regulatory systems across continents [2]. In healthcare settings, factors contributing in AMR [3] include easy access and unreasonable consumption of broad-spectrum antibiotics, inadequate guidelines for antibiotics utilization guidelines, lack of audit policies for antimicrobials, transmission of resistant strains from patient to patient and through health care providers, absence of isolation of patients colonized with resistant microbes and sub-optimal infection control measures [4]. At the moment, AMR is considered to be accountable for more than 700,000 deaths per year globally and it is anticipated that this epidemic rise will result in 10 million deaths annually by 2050, which will increase the global economic burden with an estimated cost of USD 100 trillion [5,6]. The AMR situation in Pakistan is worrisome with a recent report suggesting an antimicrobial consumption of 66.7% in hospitals and 62.2% in the community; however, no national surveillance system exists, thereby making it difficult to achieve a clear picture of AMR burden [7,8].

Antibiotic targets are usually preserved across the bacterial species and used for the development of new antibiotics [9]. β-lactams are the largest group of antibiotics that are most regularly prescribed in health care settings considering their safety, effectiveness and wide range of activity against Gram-negative and Gram-positive microorganisms [10]. Antibiotics are classified into several groups depending on their action mechanisms. Over the years, bacteria have developed several sophisticated resistance mechanisms [11]. However, the enzymatic degradation of antibiotics is considered as one of the most widely used resistance mechanisms by bacteria [9]. Many enzymes have been discovered that play a critical role in resistance emergence by degrading and modifying the function of antibiotics such as carbapenemases. Carbapenemase-encoding genes are mainly found on mobile genetic elements, hence contributing to their rapid dissemination across different bacterial species [12]. Clinically relevant core carbapenemases include KPC, NDM, OXA48, VIM and IMP [13]. Notably, carbapenem hydrolyzing enzymes reported among *Enterobacterales* from Pakistan include *bla*_KPC2_/*bla*_NDM-1_ [14], *bla*_NDM-1_/*bla*_OXA-48_ [15], *bla*_KPC2_ [16], *bla*_NDM-7_, *bla*_VIM_ and *bla*_IMP_ [17,18].

Different clinical settings facilitate differently towards the resistance development by the colonization of carbapenem-resistant *Enterobacterales* (CRE) leading to frequent outbreaks and further exhausting the depleting pool of effective antimicrobials [7]. Recently, an overall 16.5% infection risk was reported among CRE-colonized patients [19], while a 28% infection rate of CRE was reported from Egypt, consisting of 83% *Escherichia coli* (*E. coli*) and 17% *Klebsiella pneumoniae* (*K. pneumoniae*) [20]. High prevalence of CRE isolated from sink drains of health care facilities were observed recently from Pakistan [21]. Similarly, carbapenemase-producing *Enterobacterales* originating from kitchen of hematology ward were implicated in resistance transmission [22]. Global reports about nosocomial outbreaks of carbapenemase-producing strains showed its association with different clinical wards such as the VIM-producing *Enterobacter cloacae* outbreak in association with an ICU from France [23], IMP-6 CPE from Japan [24], KPC-2-producing *K. pneumoniae* from Greece and China [25,26], OXA-23-carrying carbapenem-resistant *Acinetobacter baumannii* (*A. baumannii*) in an ICU ward from China [27] and NDM-1-producing *K. pneumoniae* associated with an ICU from a Portuguese hospital and France [28,29]. Reports from Pakistan have described increasing carbapenem resistance (CR) rates up to 71% among CRE due to carbapenemases [3,30]. However, there are no data available from Pakistan that comprehensively describes the CR burden in association with clinical setup among *Enterobacterales*.

Carbapenemases usually spread through clonal lineages associated with conjugative plasmids, thus making their dispersal more convenient among nosocomial pathogens [31]. Carbapenemase-encoding genes are found associated with different sequence types such as the rapid spread of *bla*_KPC–3_-ST384 *K. pneumoniae* and *bla*_KPC–2_-ST101 among *Enterobacterales* reported from Spain [32,33,34], *bla*_KPC–2_-ST15 *K. pneumoniae* from China [35], *bla*_NDM-1_-ST307 *K. pneumoniae* from France [36], *bla*_OXA-48_-ST399 *E. coli* from the UK [37], *bla*_NDM-1_-ST147 *K. pneumoniae* from Italy [38], *bla*_NDM-1_-ST11 *K. pneumoniae* from Portugal [29], *bla*_VIM-2_-ST121 *Pseudomonas aeruginosa* (*P. aeruginosa*) from Netherlands [39] and *bla*_NDM-1_-ST11 *K. pneumoniae* from Pakistan [40]. This heterogeneous clonal background showed its importance for global dissemination of carbapenemases among *Enterobacterales*. Therefore, it is critical to assess the exposure risk and genetic profile of CRE in health care settings through surveillance to devise prevention strategies. The current study was designed to assess and characterize the CR burden in terms of antibiotic resistance profile, prevalence of antibiotic resistance genes, genetic diversity and clonality from Pakistan.

## 2. Results

### 2.1. Phenotypic Identification and Distribution of Bacterial Strains

During the study period, a total of 2170 clinical strains were collected from Mayo hospital, Lahore, Pakistan. The most prevalent species (spp.) among clinical strains were *K. pneumoniae* (*n* = 668, 30.8%) and *E. coli* (*n* = 544, 25.1%), while other genera were *Pseudomonas* (*n* = 384, 17.6%), *Proteus* (*n* = 175, 8.1%), *Acinetobacter* (*n* = 163, 7.5%), *Citrobacter* (*n* = 106, 5.0%), *Morganella* (*n* = 55, 2.5%), *Providencia* (*n* = 48, 2.2%) and *Burkholderia* (*n* = 27, 1.2%). Gender-wise categorization showed that clinical specimens were mainly obtained from males (*n* = 1288, 59.4%). The distribution of strains showed that the predominant origins of the collected specimens were general surgery units (608/2170, 28.0%), ICUs (412/2170, 19.1%) and general medicine units (360/2170, 16.6%) with wound samples (587/2170, 27.0%), pus samples (473/2170, 21.7%), blood samples (261/2170, 12.0%) and urine samples (204/2170, 9.4%) representing the main specimen types. The frequency of identified species, obtained from different specimen types and clinical wards is given in Table 1.

It was observed that wound and blood samples were significantly associated with *E. coli* (*p* < 0.001), *Pseudomonas* spp. (*p* < 0.005) and *Citrobacter* spp. (*p* = 0.020); pus samples with *K. pneumoniae* (*p* = 0.0003) and *Acinetobacter* spp. (*p* = 0.023); and urine samples with *K. pneumoniae* (*p* = 0.0003), *E. coli* (*p* < 0.0001) and *Acinetobacter* spp. (*p* = 0.020). Furthermore, the ICU was significantly associated with *K. pneumoniae* (*p* = 0.04), *E. coli* (*p* < 0.0001), *Pseudomonas* spp. (*p* = 0.0002) and *Proteus* spp. (*p* = 0.013); the nephrology ward with *K. pneumoniae* (*p* = 0.02) and *E. coli* (*p* = 0.0001); the pediatric medicine ward with *E. coli* (*p* = 0.0008), *Pseudomonas* spp. (*p* = 0.0001), *Acinetobacter* spp. (*p* = 0.019) and *Citrobacter* spp. (*p* = 0.019); and the general medicine unit with *Acinetobacter* spp. (*p* = 0.008).

### 2.2. Antimicrobial Susceptibility Trend

The antimicrobials used for susceptibility profiling of different species were selected as per criteria given by Magiorakos et al. [41]. Resistance against β-lactam combination agents, fluoroquinolones, aminoglycosides and trimethoprim/sulfamethoxazole was observed with higher susceptibility against tigecycline and polymyxin B. The following resistance rates of the antimicrobials were observed; cefazolin (1101/1294, 85.1%), cefepime (1808/2170, 83.3%), ceftazidime (1779/2170, 82.0%), cefuroxime (1285/1575, 81.6%), cefotaxime (1401/1786, 78.4%), ceftaroline (922/1212, 76.1%), ampicillin (983/1294, 76.0%), cefoxitin (1125/1490, 75.5%), aztreonam (1476/1994, 74.0%), ciprofloxacin (1572/2170, 72.4%), amoxicillin-clavulanic acid (1059/1469, 72.1%), trimethoprim-sulfamethoxazole (904/1375, 65.7%), amikacin (1072/2115, 50.7%), piperacillin-tazobactam (895/2170, 41.2%), doxycycline (485/1375, 35.5%), fosfomycin (350/928, 37.7%), ampicillin-sulbactam (63/163, 38.7%), polymyxin B (91/1215, 7.5%) and tigecycline (99/1373, 7.2%). CR was found in 42.1% (913/2170) of isolates and 57.9% (1257/2170) were carbapenem susceptible. Higher CR rates were detected among *K. pneumoniae* (309/913, 33.8%) and *E. coli* (223/913, 24.4%), followed by *Pseudomonas* spp. (169/913, 18.5%), *Acinetobacter* spp. (67/913, 7.3%), *Proteus* spp. (61/913, 6.9%), *Citrobacter* spp. (45/913, 4.9%), *Providencia* spp. (19/913, 2.1%), *Morganella* spp. (15/913, 1.6%) and *Burkholderia* spp. (5/913, 0.5%). CR was significantly associated with *K. pneumoniae* (*p* = 0.008) and *Proteus* spp. (*p* = 0.043). The details of antimicrobial susceptibility trends among individual species are given in Table 2.

The CR burden was analyzed in clinical wards and specimen types. The prevalence of CR among clinical specimens was higher in wound samples (292/913, 32.0%), pus samples (206/931, 22.6%), urine samples (103/913, 11.3%) and blood samples (97/931, 10.6%), while the general surgery unit (262/913, 28.7%), general medicine unit (180/913, 19.7%) and ICU (157/913, 17.2%) were the dominant hospital sections involved in the CR spread. It was observed that the occurrence of CR was statistically significant among wound samples (*p* = 0.00001), urine samples (*p* = 0.01), tissue samples (*p* = 0.00001) and tip cell samples (*p* = 0.037). Additionally, the general medicine unit (*p* = 0.0008) and oncology ward (*p* = 0.006) were significantly associated with CR. The results are given in Table 3.

### 2.3. Prevalence of Antimicrobial Resistance Genes

Carbapenemase production was found in 86.4% (789/913) of isolates with *K. pneumoniae* (283/789, 35.9%), *E. coli* (199/789, 25.2%), *Pseudomonas* spp. (145/789, 18.4%), *Proteus* spp. (53/789, 6.7%), *Acinetobacter* spp. (49/789, 6.2%), *Citrobacter* spp. (31/789, 3.9%), *Morganella* spp. (12/789, 1.5%), *Providencia* spp. (13/789, 1.6%) and *Burkholderia* spp. (4/789, 0.5%). On the other hand, 13.6% (124/913) carbapenem-resistant strains were non-carbapenemase-producing, pointing towards the involvement of alternative resistance mechanisms for carbapenem-resistant phenotypes in this study population.

Carbapenemase-encoding genes were detected in 82.2% (649/789) of carbapenemase-producing isolates with 15.0% (97/649) coharbored genes and 85.0% (552/649) single genes. The frequency of carbapenemase resistance genes among detected species was 36.0% (234/649) *K. pneumoniae*, 22.0% (143/649) *E. coli*, 20.5% (133/649) *Pseudomonas* spp., 7.2% (47/649) *Acinetobacter* spp., 7.1% (46/649) *Proteus* spp., 4.0% (26/649) *Citrobacter* spp., 1.7% (11/649) *Providencia* spp., 1.0% (6/649) *Morganella* spp. and 0.5% (3/649) *Burkholderia* spp. The detected carbapenemases were *bla*_NDM-1_ 41.1% (267/649), *bla*_OXA-48_ 32.6% (212/649), *bla*_KPC-2_ 5.5% (36/649), *bla*_VIM_ 3.0% (19/649), *bla*_IMP_ 2.7% (18/649), *bla*_NDM-1_/*bla*_OXA-48_ 11.4% (74/649), *bla*_OXA-48_/*bla*_VIM_ 3.1% (20/649), *bla*_OXA-48_/*bla*_IMP_ 0.1% (1/649) and *bla*_VIM_/*bla*_IMP_ 0.3% (2/649). Among carbapenemase gene-positive strains, 14.2% (92/649) were XDR and 85.8% (557/649) MDR. The results are given in Table 4.

The distribution of detected carbapenemases was analyzed in relation to clinical wards and specimens. It was observed that *bla*_KPC-2_ was significantly associated with the general surgery unit (16/36, 44.4%, *p* = 0.029); *bla*_NDM-1_ with wound samples (82/267, 30.7%, *p* = 0.041), ICU (74/267, 27.7%, *p* < 0.001), cardiology ward (9/267, 3.4%, *p* = 0.042) and pediatric medicine ward (9/267, 3.4%, *p* = 0.013); *bla*_OXA-48_ with tip cell samples (9/212 4.2%, *p* = 0.041), general surgery unit (43/212, 20.3%, *p* = 0.001), ICU (23/212, 10.8%, *p* < 0.001), general medicine unit (59/212, 27.8%, *p* = 0.005), nephrology ward (3/212, 1.4%, *p* = 0.020), cardiology ward (19/212, 9.0%, *p* = 0.008), pediatric medicine ward (23/212, 10.8%, *p* < 0.001) and orthopedic surgery ward (13/212, 6.1%, *p* = 0.007); *bla*_VIM_ with tracheal secretion samples (5/19, 26.3%, *p* < 0.001), tip cell samples (2/19, 10.5%, *p* = 0.021) and oncology ward (3/19, 15.8%, *p* < 0.001); *bla_I_*_MP_ with pus samples (9/18, 50.0%, *p* = 0.006), CV line samples (2/18, 11.1%, *p* < 0.001) and chest medicine ward (3/18, 16.7%, *p* = 0.001); *bla*_NDM-1_/*bla*_OXA-48_ with wound samples (38/74, 51.4%, *p* = 0.002), blood samples (13/74, 17.6%, *p* = 0.012), urine samples (1/74, 1.4%, *p* = 0.003) and general surgery ward (36/74, 48.6%, *p* < 0.001); *bla*_OXA-48_/*bla*_VIM_ with urine samples (9/20, 45.0%, *p* < 0.001) and nephrology ward (7/74, 35.0%, *p* < 0.001). The results are given in Table 5.

ESBL-producer strains were 89.9% (821/913) of the samples, and ESBL resistance genes were found in 92.4% (759/821) of isolates. The prevalence of detected ESBL resistance genes was as follows: *bla*_SHV_ 53.3% (405/759), *bla*_CTX-M_ 61.8% (469/759), *bla*_TEM_ 39.1% (297/759), *bla*_SHV_/*bla*_CTX-M_ 46.7% (355/759), *bla*_SHV_/*bla*_TEM_ 22.3% (169/759), *bla*_CTX-M_/*bla*_TEM_ 21.3% (162/759) and *bla*_SHV_/*bla*_CTX-M_/*bla*_TEM_ 18.6% (141/759).

### 2.4. Genetic Diversity Analysis

Further, the genetic diversity of *K. pneumoniae* strains harboring *bla*_NDM-1_ (*n* = 83), *bla*_KPC-2_ (*n* = 36) and *bla*_NDM-1_/*bla*_OXA-48_ (*n* = 37) and *E. coli* strains harboring *bla*_NDM-1_ (*n* = 68) and *bla*_NDM-1_/*bla*_OXA-48_ (*n* = 13) were accessed in terms of clonal lineage and plasmid content. The sequence types identified among *K. pneumoniae* were ST147 (39.7%, 62/156), ST258 (10.9%, 17/156), ST11 (23.0%, 36/156), ST14 (15.4%, 24/156) and ST340 (9.6%, 15/156), and among *E. coli* were ST131 (21.2%, 18/81), ST405 (34.5%, 28/81), ST101 (19.7%, 16/81), ST69 (7.4%, 6/81) and ST10 (16.0%, 13/81). Plasmid replicon types IncFII, IncA/C, IncN, IncL/M, IncFIIA and IncFIIK were observed. The detailed results are given in Table 6.

It was observed that different carbapenemases were present on different sequence types, depicting the adaptability of sequence types towards carbapenemases such as *bla*_KPC-2_-ST147 (16/156, 10.2%), *bla*_NDM-1_-ST147 (35/156, 22.4%), *bla*_NDM-1_/*bla*_OXA-48_-ST147 (17/156, 11.1%), *bla*_KPC-2_-ST258 (10/156, 6.4%), *bla*_NDM-1_/*bla*_OXA-48_-ST258 (7/156, 4.5%), *bla*_NDM-1_-ST340 (8/156, 5.1%), *bla*_KPC-2_-ST11 (10/156, 6.4%), *bla*_NDM-1_-ST11 (22/156, 14.1%), *bla*_NDM-1_/*bla*_OXA-48_-ST11 (13/156, 8.3%) and *bla*_NDM-1_-ST14 (18/156, 11.5%) among *K. pneumoniae* strains, and *bla*_NDM-1_-ST405 (22/81, 27.2%), *bla*_NDM-1_/*bla*_OXA-48_-ST405 (8/81, 9.8%), *bla*_NDM-1_-ST131 (9/81, 11.1%), *bla*_NDM-1_/*bla*_OXA-48_-ST131 (1/81, 1.2%), *bla*_NDM-1_-ST101 (16/81, 19.7%), *bla*_NDM-1_/*bla*_OXA-48_-ST101 (2/81, 2.5%), *bla*_NDM-1_-ST69 (10/81, 12.3%), *bla*_NDM-1_/*bla*_OXA-48_-ST69 (1/81, 1.2%), *bla*_NDM-1_-ST10 (11/81, 13.6%) and *bla*_NDM-1_/*bla*_OXA-48_-ST10 (1/81, 1.2%) among *E. coli* strains.

## 3. Discussion

Carbapenem resistance is considered as one of the critical threats associated with hospital-acquired infections, especially in developing countries. Therefore, timely surveillance efforts are required to reduce the spread of CRE [42]. The current study was designed to characterize the key determinants for resistance spread in a tertiary care hospital.

Our results showed that the patients were infected mostly with *K. pneumoniae* and *E. coli* strains while infrequently detected genera were *Pseudomonas, Proteus*, *Acinetobacter*, *Citrobacter*, *Morganella*, *Providencia* and *Burkholderia*. Previous studies from Pakistan showed that *K. pneumoniae* and *E. coli* were the most commonly detected pathogens responsible for nosocomial infections [43,44,45]. While the global data suggested the higher prevalence of *K. pneumoniae*, *P. aeruginosa*, *E. coli* and *A. baumannii* from Tanzania, Algeria, Nepal and Saudi Arabia [46,47,48,49], our study demonstrated 42.1% CR among *Enterobacterales*. This is higher than prevalences previously reported from Pakistan such as 21.84% [50], 25.5% [51], 9.6% [52] and 6.5% [45]. However, our results are in agreement with the global reports that CRE prevalence is at alarming rates, such as 65.0% from the USA [53], 42.6% from Cuba [54] and 34.3% from China [55]. We observed that *K. pneumoniae* is the leading CRE pathogen, accounting for 33.8% of the total CR load, followed by *E. coli* (24.4%) and *Pseudomonas* spp. (18.5%). *E. coli* and *K. pneumoniae* were considered the prime reason for CRE as evidenced by a number of other studies; *E. coli* (86.0–38.24%) [17,45,51,56] and *K. pneumoniae* (60.0–31.62%) [15,45,56,57]). We observed that CR is significantly associated with *K. pneumoniae* (*p* = 0.008) and *Proteus* spp. (*p* = 0.043). Similarly, another study reported significant relation of CR with *K. pneumoniae* (800/1499, 53.0%, *p* = 0.0008) [58].

The National AMR action Plan for Pakistan 2017–2018 suggested a rate of 30% CR in *K. pneumoniae*, while much lower CR rates were reported in *P. aeruginosa isolates* (6.5%) [8]. However, another study showed higher CR rates among *Pseudomonas* spp. (34.0%) but lower rates among *E. coli* (7.0%) and *Klebsiella* spp. (8.0%) [52]. These reports together with our data suggested that the CR trend among *Pseudomonas* spp. is changing with time and a notable CR increase was observed from Pakistan, as can be seen in the studies showing 24.2% in 2012 [59], 49.5% imipenem resistance in 2015 [60], 81.6% in 2019 [61], 43.2% in 2020 [62] and 66.4% meropenem resistance in 2022 [63]. In contrast to our results of *Acinetobacter* spp. (7.3%), data from Pakistan suggested a sharp increase of CR among *Acinetobacter* spp. from 50% in 2011 to 95.5% in 2015 [64,65,66], 61.89% imipenem resistant *Acinetobacter* spp. in 2018 and 84.0% in 2022 [56,67]. High CR rates among *Acinetobacter* spp. and *Pseudomonas* spp. are alarming in Pakistan as these species exhibit intrinsic resistance to many antibiotics, leaving few therapeutic choices available. Our results strengthen the WHO recommendations for both species as critical pathogens [30,68].

On the other hand, CR among other species observed in our study suggested lower resistance rates, including *Proteus* spp. (6.9%), *Citrobacter* spp. (4.9%), *Providencia* spp. (2.1%), *Morganella* spp. (1.6%) and *Burkholderia* spp. (0.5%). Our results are in accordance with other studies with results such as *Proteus* spp. (3.0%), *Citrobacter* spp. (1.0%) [52], *Morganella morganii* (*M. morganii)* (1.5%), *Proteus mirabilis* (6.5%), *Citrobacter freundii* (*C. freundii)* (4.5%) [69], *Morganella* spp. (0.5%) [70] and *C. freundii* (41.6%) and *M. morganii* (3.0%) [71,72]. However, no report is available about CR among *Providencia* spp. and *Burkholderia* spp. from Pakistan as per our knowledge. The trend of CR in our study population suggests that infections are mostly treated empirically by using broad-spectrum antimicrobials without proper testing in developing countries, thereby promoting resistant phenotypes.

Global reports demonstrated variation in the dissemination of CRE such as 52.0% CRE from Vietnam with *K. pneumoniae* (69.0%) and *E. coli* (59.0%) as prevalent species [73], 12.4% CRE from Indonesia [74], 2.9% CRE from Korea [75], 77.8% CRE from India [76], 54.1% CRE from Egypt with CR *K. pneumoniae* (53.7%) and *E. coli* (27.1%) [77], 22.0% CRE from Nigeria with CR *K. pneumoniae* (35.9%), *P. aeruginosa* (30.8%) [78]. While a European cohort study reported 55.0% (944/1717) CRE [79]. Surveillance data by ECDC on AMR showed that CR has increased in Greece with 64.7% presence in *K. pneumoniae* and 63.9% *E. coli* [80]. Interestingly, identification of CRE from Japan is still scarce with 0.5% meropenem resistance in *K. pneumoniae* [24]. Similarly, much lower CR among *E. coli* (0.02%) and *K. pneumoniae* (0.18%) reported from Netherlands [81]. On the other hand, variable range of imipenem resistance in *P. aeruginosa* was observed worldwide, including in China (33.2%), India (29.6%), Japan (8.0%), Italy (28.5%), Turkey (43.3%), Ukraine (54.7%), United States (21.4%) and Kuwait (44.7%) [82]. From Romania, 6.25% *Proteus* spp. and 45.79% *Providencia* spp. were carbapenem resistant [83].

In our study, wound (32.0%) and pus (22.6%) were the predominant specimens for CRE isolation, while clinical wards with higher proportions of CRE were general surgery (28.7%), general medicine (19.7%) and ICU (17.2%). However, tracheal aspirate (25.0%), urine (24.26%), pus (25.53%), and surgical units (51.4%), ICU (65.3%), medical units (43.5%), pediatric wards (71.4%) were the previously reported causes of CRE infections in Pakistan [45,52]. Worldwide reports established that ICU-related colonization of CRE is higher, with results such as 86.15%, 35.5%, 31.0%, 24.0% and 12.3%, thus favoring the resistance selection process [54,83,84,85,86]. The Greek System for the Surveillance of Antimicrobial Resistance reported that CR increased from <1% in 2001 to 42% in medical wards and to 72% in ICUs among *K. pneumoniae* isolates [25]. Furthermore, respiratory-, surgical- and urinary-associated healthcare CRE infections increased from 5% to 25% in developed countries [87,88]. The most frequent source of CRE infection included urinary tract (36.2%), followed by blood (26.3%) and surgical wound (17.1%) [54,84]. Our study described the significant association of wound (*p* = 0.00001), urine (*p* = 0.01), tissue (*p* = 0.00001) and tip cell samples (*p* = 0.037) with CR, while general medicine units (*p* = 0.0008) and oncology wards (*p* = 0.006) remained statistically significant in relation to CR spread. Another study from Pakistan reported association of wound infections with *Acinetobacter* spp. (OR = 1.79) and *Pseudomonas* spp. (OR = 1.29) [56]. Urine was found to be the most common origin of CRE from the USA (*p* < 0.0001) [58] and Egypt (*p* = 0.035) [20]. Therefore, the current investigation highlights the constant requirement of containment plans in healthcare departments associated with CR to prevent and slow the process of its expansion.

Enzyme-mediated CR accounts for 20–70% of the total AMR burden among *Enterobacterales* thereby highlighting carbapenemase production as the most common mode of resistance [89]. A total of 86.4% carbapenemase-producing *Enterobacterales* (CPE) were identified in present study with 35.9% *K. pneumoniae*, 25.2% *E. coli* and 18.4% *Pseudomonas* spp. as main producer species. Other reports from Pakistan supplement our findings that *K. pneumoniae* and *E. coli* were major contributors of the total carbapenemase production among *Enterobacterales* [15,17,40,44,57,90,91]. Another study observed a high proportion of carbapenemase production among *Citrobacter* spp. (66%), *Acinetobacter* spp. (53%), *Pseudomonas* spp. (51%) and *Proteus* spp. (20%) [52]. In contrast, our results indicated lower rates among *Proteus* spp. (6.7%), *Acinetobacter* spp. (6.2%) and *Citrobacter* spp. (3.9%). However, we observed carbapenemase production among *Morganella* spp. (1.5%), *Providencia* spp. (1.6%) and *Burkholderia* spp. (0.5%) for the first time from Pakistan.

The key contributing carbapenemases involved in the expansion of CPE in the study population are *bla*_NDM-1_ and *bla*_OXA-48_, confirming the existing data from Pakistan [14,15,17,40,45,57,90,91,92]. We observed a considerable increase in the prevalence of KPC-producing *K. pneumoniae* (15.4%). It is noteworthy that first KPC was detected from Pakistan in 2016; afterwards, few reports emerged since 2020 describing the 1.8–17.6% prevalence of *bla*_KPC-2_ [14,16,45,93]. Among *Pseudomonas* spp., we detected *bla*_VIM_ (8.3%) and *bla*_OXA-48_/*bla*_VIM_ (6.7%), while previously 2.3%–42.3% *bla*_VIM_ prevalence was described [62,94,95]. We detected *bla*_IMP_ more frequently in *Pseudomonas* spp. along with one report in *Proteus* spp. However, *bla*_VIM_ and *bla*_IMP_ were reported in *Acinetobacter* spp. previously from Pakistan [56,94,95,96]. Another important finding of our study was the emergence of *bla*_NDM-1_ (*n* = 2), *bla*_OXA-48_ (*n* = 3) and *bla*_IMP_ (*n* = 1) in *Morganella* spp., while only report available from Pakistan recorded *bla*_NDM-1_ (*n* = 2) in *M. morganii* [69]. Furthermore, this is the first report that detected *bla*_NDM-1_ (*n* = 1) and *bla*_OXA-48_ (*n* = 2) in *Burkholderia* spp. and the coexistence of *bla*_NDM-1_/*bla*_OXA-48_ (*n* = 2) in *Providencia* spp. We observed a significant association of general surgery units with *bla*_KPC-2_ (*p* = 0.029), *bla*_OXA-48_ (*p* = 0.001) and *bla*_NDM-1_/*bla*_OXA-48_ (*p* < 0.001); ICU with *bla*_NDM-1_ (*p* < 0.001) and *bla*_OXA-48_ (*p* < 0.001); cardiology and pediatric medicine wards with *bla*_NDM-1_ (*p* = 0.042, *p* = 0.013) and *bla*_OXA-48_ (*p* = 0.008, *p* < 0.001); general medicine units with *bla*_OXA-48_ (*p* = 0.005); nephrology wards with *bla*_OXA-48_ (*p* = 0.020) and *bla*_OXA-48_/*bla*_VIM_ (*p* < 0.001); wound samples with *bla*_NDM-1_ (*p* = 0.041) and *bla*_NDM-1_/*bla*_OXA-48_ (*p* = 0.002); urine samples with *bla*_NDM-1_/*bla*_OXA-48_ (*p* = 0.003) and *bla*_OXA-48_/*bla*_VIM_ (*p* < 0.001); and blood samples with *bla*_NDM-1_/*bla*_OXA-48_ (*p* = 0.012). We could not find another association study from Pakistan.

The main reason for the emergence of different STs globally is the ability of strains to disseminate carbapenemases through plasmids and their successful adaption to different healthcare environments. Our data revealed that successful high-risk clones of *K. pneumoniae* and *E. coli* have emerged in Pakistan, such as *bla*_KPC-2_-ST147, *bla*_NDM-1_-ST147, *bla*_NDM-1_/*bla*_OXA-48_-ST147, *bla*_KPC-2_-ST258, *bla*_NDM-1_/*bla*_OXA-48_-ST258, *bla*_NDM-1_-ST340, *bla*_KPC-2_-ST11, *bla*_NDM-1_-ST11, *bla*_NDM-1_/*bla*_OXA-48_-ST11 and *bla*_NDM-1_-ST14 among *K. pneumoniae*, and *bla*_NDM-1_-ST405, *bla*_NDM-1_/*bla*_OXA-48_-ST405, *bla*_NDM-1_-ST131, *bla*_NDM-1_/*bla*_OXA-48_-ST131, *bla*_NDM-1_-ST101, *bla*_NDM-1_/*bla*_OXA-48_-ST101, *bla*_NDM-1_-ST69, *bla*_NDM-1_/*bla*_OXA-48_-ST69, *bla*_NDM-1_-ST10 and *bla*_NDM-1_/*bla*_OXA-48_-ST10 among *E. coli*. The previously described STs from Pakistan include *bla*_KPC-2_-ST258, *bla*_NDM-1_-ST147, *bla*_NDM-1_/*bla*_OXA-48_-ST147, *bla*_NDM-1_-ST11, *bla*_NDM-1_/*bla*_OXA-48_-ST405, *bla*_NDM-7_/*bla*_OXA-48_-ST405, *bla*_NDM-1_-ST405, *bla*_NDM-7_/*bla*_OXA-48_-ST131, *bla*_NDM-1_/*bla*_OXA-48_-ST131, *bla*_NDM-1_-ST131, *bla*_NDM-1_-ST10, *bla*_NDM-1_/*bla*_OXA-48_-ST101, *bla*_NDM-1_-ST101, *bla*_NDM-1_/*bla*_OXA-48_-ST648, *bla*_OXA-48_-ST231 and *bla*_NDM-1_-ST859 [15,16,17,97]. Furthermore, we observed the emergence of *bla*_KPC-2_-ST147, *bla*_NDM-1_-ST340 and *bla*_NDM-1_-ST14 in *K. pneumoniae* and *bla*_NDM-1_-ST69 and *bla*_NDM-1_/*bla*_OXA-48_-ST69 in *E. coli*.

## 4. Conclusions

In this study, we reported the detailed analysis of carbapenem resistance burden and the emergence of *bla*_KPC-2_-ST147, *bla*_NDM-1_-ST340 and *bla*_NDM-1_-ST14 in *K. pneumoniae* isolates, and *bla*_NDM-1_-ST69 and *bla*_NDM-1_/*bla*_OXA-48_-ST69 in *E. coli* isolates coharboring ESBLs from Pakistan. Moreover, we described *bla*_NDM-1_ (*n* = 1) and *bla*_OXA-48_ (*n* = 2) in *Burkholderia* spp. and the coexistence of *bla*_NDM-1_/*bla*_OXA-48_ (*n* = 2) in *Providencia* spp. for first time in the study population. Our data indicated that the lack of antimicrobial stewardship and misuse augmented by diagnostic difficulties in developing countries are accelerating the evolution and spread of high-risk STs and hyper-efficient plasmids. This situation is miserable, especially in healthcare settings with immense antimicrobial selection pressure, thereby highlighting the expansion of high-risk clones as a resistance reservoir.

## 5. Methodology

**Figure 1:**
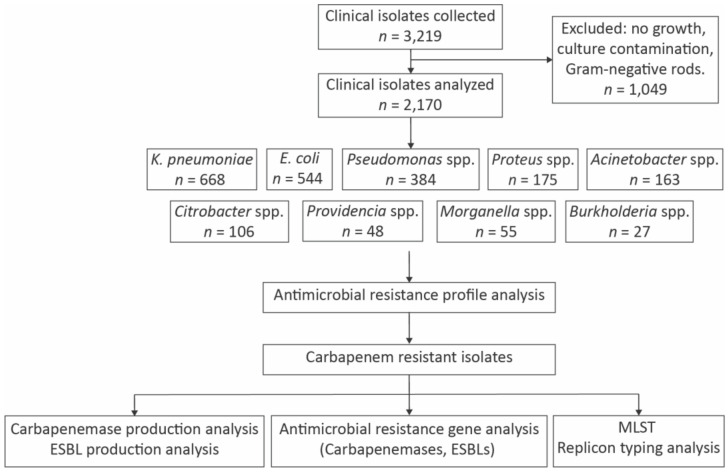
Flow diagram of clinical isolates processing.

The clinical strains were collected between May 2019 and April 2022 from the routine diagnostic laboratory, Mayo hospital, Lahore, Pakistan. Mayo hospital is one of the largest hospitals in South East Asia with a 3000 bed capacity. The clinical isolates were processed as given in Figure 1. Clinical specimens were phenotypically characterized by analyzing colony morphology and Grams staining by culturing on MacConkey agar and cysteine lactose electrolyte-deficient media (Oxoid Ltd., Basingstoke, UK) for urine samples. Biochemical characterization was performed by API-20E and API-20NE (BioMerieux, Marcy-IEtoile, France).

### 5.1. Antimicrobial Susceptibility Testing

Antimicrobial susceptibility testing was performed by standard Kirby–Bauer disc diffusion method using Mueller–Hinton agar (Oxoid, Ltd., Basingstoke, UK), according to the “Performance Standards for Antimicrobial Disk Susceptibility Tests; CLSI Supplement M100, 30th Edition”. The following antibiotic disks were used: imipenem (10 μg), meropenem (10 μg), cefazolin (30 μg), cefuroxime (30 μg), ceftazidime (30 μg), cefotaxime (30 μg), cefepime (30 μg), cefoxitin (30 μg), ceftaroline (30 μg), ampicillin (10 μg), amoxicillin-clavulanic acid (20/10 μg), aztreonam (30 μg), ciprofloxacin (5 μg), trimethoprim-sulfamethoxazole (1.25/23.75 μg), tigecycline (15 μg), fosfomycin (50 μg), polymyxin-B (300 U), doxycycline (30 μg), amikacin (10 μg), piperacillin-tazobactam (100/10 μg), ampicillin-sulbactam (20 μg) (Oxoid, Ltd., Basingstoke, UK). For polymyxin B, the standard broth microdilution method was used as per CLSI recommendation (MIC breakpoints; intermediate ≤ 2, resistant ≥ 4). For tigecycline, EUCAST breakpoints were used [98]. Quality control strains were *E. coli* ATCC 25922 and *P. aeruginosa* ATCC 27853.

Carbapenemase-producing strains were identified by using the modified carbapenem inactivation method (mCIM) [99]. Briefly, 1 or 2 colonies of bacterial growth were mixed with 2 mL of tryptone soy broth (TSB media; ThermoFischer Scientific, Waltham, MA, USA). Meropenem antibiotic disc was added into the bacterial suspension under sterile conditions and incubated at 35 ± 2 °C for 4 h. Meanwhile, a suspension of the mCIM indicator organism *E. coli* ATCC 25922 (carbapenem-sensitive strain) with turbidity equivalent to 0.5 McFarland standard was prepared and inoculated on a Mueller–Hinton agar (Oxoid, UK) plate. The meropenem antibiotic disc from cultured TSB bacterial suspension was transferred to inoculate the MHA plate with indicator strain. Plates were dried for 3–10 min before adding the meropenem antibiotic disc. *K. pneumoniae* ATCC BAA-1705 strain was used as quality control strain. The plate was incubated for 18 to 24 h at 35 ± 2 °C. ESBL producer strains were identified by CHROMagar^TM^ ESBL media (CHROMagar, Paris, France).

### 5.2. Antimicrobial Resistance Gene Analysis

The heat lysis method was used for genomic DNA extraction [100]. In short, 2 to 3 bacterial colonies were mixed with 500 μL sterile dH2O in 1.5 mL microcentrifuge tube. The sample was incubated at 98 °C for 10 min/300 rpm in thermomixer (FischerScientific, Waltham, MA, USA). Sample was centrifuged at 1000 rpm for 10 min and supernatant containing DNA was collected in a new tube. DNA was stored at −80 °C until further processing. Carbapenemase resistance genes (*bla*_KPC-2_, *bla*_NDM-1_, *bla*_VIM_, *bla*_IMP_, *bla*_OXA-48_) and selected ESBLs (*bla*_SHV_, *bla*_TEM_ and *bla*_CTX-M_) were detected by standard PCR. The PCR reaction mixture contained 25 μL of 2 × PCR Master Mix (catalogue # K0171, Thermoscientific, Waltham, MA, USA), 10 μM of each primer, 0.5 ng of DNA and dH2O up to 50 μL in a thermal cycler (Proflex, ABI, Haines City, FL, USA). Amplicons were resolved by agarose gel electrophoresis (1–1.5%). The primer sequences and PCR cycling conditions are given in Table S1.

### 5.3. Allele Identification by Sequencing

Sanger’s sequencing method was used for the *bla*_NDM_ and *bla*_KPC_ allele identification. BigDye terminator v3.1 kit was used for cycle sequencing as per kit instructions. Briefly, 10 μL PCR reaction mixture contained BigDye terminator 3.1 Ready Reaction Mix 4 μL, forward primer (3.2 pmol) 0.5 μL, purified DNA template (5–20 ng) 2 μL and dH2O 3.5 μL. PCR cycling conditions were 96 °C 1 min, 96 °C 10 s, 50 °C 5 s, 60 °C 2 min (35 cycles). PCR product was purified by using BigDye XTerminator purification kit as per kit instructions and capillary electrophoresis was performed by Genetic Analyzer (ABI-3500, Thermo Fischer, Waltham, MA, USA). Sequencing analysis software v6.1 and basic local alignment tool (BLAST, NCBI) were used for data analysis and interpretation. CLC Sequence Viewer 7 was used for sequence alignment and mutation analysis.

### 5.4. Determination of Genetic Diversity by Multilocus Sequence Typing and Plasmid Replicon Typing

*K. pneumoniae* and *E. coli* strains harboring *bla*_NDM-1_, *bla*_KPC_ and *bla*_NDM-1_/*bla*_OXA-48_ were further subjected to multilocus sequence typing (MLST) analysis. For *K. pneumoniae*, seven housekeeping genes were used [101]: glyceraldehyde-3-phosphate dehydrogenase A gene (*gapA*), translation initiation factor IF-2 gene (*infB*), malate dehydrogenase gene (*mdh*)*,* phosphoglucose isomerase gene (*pgi*)*,* phosphoporin E gene (*phoE*), periplasmic energy transducer gene (*tonB*), beta-subunit of RNA polymerase gene (*rpoB*). For *E. coli*, eight housekeeping genes were used: DNA polymerase (*dinB*), isocitrate dehydrogenase (*icdA*), p-aminobenzoate synthase (*pabB*), polymerase PolII (*polB*), proline permease (*putP*), tryptophan synthase subunit A (*trpA*), tryptophan synthase subunit B (*trpB*) and beta-glucuronidase (*uidA*) [102]. Sequencing analyses were performed as described above by using primer sequences given in Table S1. Sequence types for *K. pneumoniae* were assigned using the MLST database (http://bigsdb.pasteur.fr/klebsiella/ (accessed on 3 March 2022)) and for *E. coli*, also the MLST database (https://pubmlst.org/bigsdb?db=pubmlst_mlst_seqdef (accessed on 11 August 2022)). Plasmid DNA was extracted from single colony of CRKP by using the plasmid isolation kit (ThermoFischer Scientific, Waltham, MA, USA). Plasmids were classified according to their incompatibility groups by using the PCR-based replicon typing method as described before [103].

### 5.5. Statistical Analysis

All statistical analyses were performed by using Statistical Package for Social Sciences software (SPSS 26). Categorical data are presented as frequency and percentage. The chi-square test was used to compare the categorical data among groups. *p*-value ≤ 0.05 was considered as significant.

## Figures and Tables

**Table 1:** Distribution of different species among clinical specimens and wards.

	*K. pneumoniae*	*E. coli*	*Pseudomo-* *nas* spp.	*Acinetob-* *acter* spp.	*Citrobac-* *ter* spp.	*Proteus* spp.	*Morgane-* *lla* spp.	*Provide-ncia* spp.	*Burkhold-* *eria* spp.
**Clinical specimens *n* (%)**									
Wound	187 (28.1)	106 (19.5)	126 (32.8)	40 (24.5)	39 (36.8)	56 (32.0)	13 (23.6)	13 (27.1)	7 (26.0)
Pus	114 (17.1)	111 (20.4)	96 (25.0)	47 (28.8)	21 (19.8)	47 (26.9)	14 (25.4)	18 (37.5)	5 (18.5)
Blood	92 (13.8)	87 (16.1)	29 (7.6)	18 (11.0)	9 (8.5)	15 (8.6)	5 (9.1)	2 (4.2)	4 (14.8)
Urine	37 (5.5)	105 (19.3)	26 (6.8)	7 (4.3)	4 (3.8)	8 (4.6)	5 (9.1)	5 (10.4)	7 (26.0)
Sputum	45 (6.7)	30 (5.5)	18 (4.7)	8 (4.9)	8 (7.5)	5 (2.6)	8 (14.5)	2 (4.2)	3 (11.1)
Tracheal secretion	43 (6.4)	21 (3.7)	10 (2.6)	13 (8.0)	2 (1.9)	7 (4.0)	-	4 (8.3)	1 (3.7)
ETT	40 (6.1)	10 (1.8)	14 (3.6)	3 (1.8)	8 (7.5)	3 (1.7)	-	2 (4.2)	-
Tissue	30 (4.5)	20 (3.7)	11 (2.9)	5 (3.1)	4 (3.8)	2 (1.1)	-	2 (4.2)	-
Tip cells	20 (3.1)	13 (2.4)	19 (5.0)	4 (2.5)	1 (0.9)	10 (5.7)	3 (5.5)	-	-
Drain	23 (3.4)	15 (2.7)	13 (3.4)	4 (2.5)	3 (2.8)	4 (2.3)	4 (7.3)	-	-
Pleural fluid	18 (2.7)	16 (3.1)	13 (3.4)	6 (3.7)	3 (2.8)	9 (5.1)	1 (1.8)	-	-
CV line	19 (2.8)	10 (1.8)	9 (2.3)	8 (4.9)	4 (3.8)	9 (5.1)	2 (3.6)	-	-
Total	668	544	384	163	106	175	55	48	27
**Clinical wards *n* (%)**									
General surgery	182 (27.2)	141 (26.0)	120 (31.3)	41 (25.2)	24 (22.6)	55 (31.4)	20 (36.4)	18 (37.5)	7 (25.9)
ICU	144 (21.5)	139 (25.5)	47 (12.2)	24 (14.7)	14 (13.2)	21 (12.0)	12 (21.8)	6 (12.5)	5 (18.5)
General medicine	104 (15.6)	91 (16.7)	60 (15.6)	39 (23.9)	14 (13.2)	30 (17.1)	10 (18.2)	7 (14.6)	5 (18.5)
Dermatology	60 (9.0)	17 (3.1)	33 (8.6)	11 (6.7)	11 (10.4)	16 (9.1)	-	1 (2.1)	-
Pediatric medicine	44 (6.7)	14 (2.6)	39 (10.2)	3 (1.8)	12 (11.3)	12 (6.9)	-	3 (6.3)	4 (14.8)
Cardiology	28 (4.2)	37 (6.8)	19 (4.9)	12 (7.4)	7 (6.6)	8 (4.6)	4 (7.3)	3 (6.3)	3 (11.1)
Chest medicine	46 (6.9)	17 (3.1)	19 (4.9)	8 (4.9)	6 (5.7)	5 (2.9)	5 (9.1)	2 (4.2)	-
Nephrology	20 (3.0)	41 (7.5)	15 (3.9)	6 (3.7)	4 (3.8)	8 (4.6)	-	2 (4.2)	3 (11.1)
Orthopedic surgery	28 (4.2)	19 (3.5)	17 (4.4)	7 (4.3)	5 (4.7)	10 (5.7)	4 (7.3)	2 (4.2)	-
Oncology	8 (1.2)	25 (4.6)	9 (2.3)	7 (4.3)	4 (3.8)	5 (2.9)	-	2 (4.2)	-
Neurology	4 (0.6)	3 (0.5)	6 (1.0)	5 (3.1)	5 (4.7)	5 (2.9)	-	2 (4.2)	-
Total	668	544	384	163	106	175	55	48	27

**Table 2:** Antimicrobial resistance profile of detected species.

	*K. pneumoniae*	*E. coli*	*Pseudomonas* spp.	*Acinetobacter* spp.	*Citrobacter* spp.	*Proteus* spp.	*Providencia* spp.	*Morganella* spp.	*Burkholderia* spp.
**Antibiotics *n* (%)**									
IMP/MEM	309 (46.2)	223 (41.1)	169 (44.0)	67 (41.1)	45 (42.4)	61 (34.8)	19 (34.5)	15 (31.2)	5 (18.5)
CFZ	569 (85.2)	473 (86.9)	-	-	-	-	36 (65.4)	-	23 (85.1)
CXM	592 (88.6)	438 (80.5)	-	-	78 (73.6)	123 (70.3)	31 (56.4)	-	23 (85.1)
CAZ	563 (84.3)	440 (80.8)	357 (92.9)	153 (93.8)	73 (68.8)	129 (73.7)	37 (67.3)	23 (47.9)	24 (88.9)
CTX	547 (81.8)	442 (81.2)	-	145 (88.9)	67 (63.2)	119 (68.0)	37 (67.3)	23 (47.9)	21 (77.7)
FEP	573 (85.8)	441 (81.0)	367 (95.5)	145 (88.9)	73 (68.8)	121 (69.1)	36 (65.4)	29 (60.4)	23 (85.1)
FOX	527 (78.8)	438 (80.5)	-	-	-	93 (53.1)	36 (65.4)	31 (64.6)	-
CPT	483 (72.3)	439 (80.7)	-	-	-	-	-	-	-
AMP	598 (89.5)	479 (88.0)	-	-	-	-	47 (85.4.0)	-	25 (92.6)
AMC	581 (87.0)	487 (89.5)	-	-	-	116 (66.3)	43 (78.1)	-	24 (88.8)
ATM	475 (71.1)	469 (86.2)	344 (89.6)	-	57 (53.7)	113 (64.6)	33 (60.0)	21 (43.7)	24 (88.8)
CIP	467 (70.0)	402 (73.9)	346 (90.1)	135 (82.8)	49 (46.2)	97 (55.4)	37 (67.3)	25 (52.0)	21 (77.7)
SXT	392 (58.7)	354 (65.1)	-	158 (96.9)	-	-	-	-	-
TGC	36 (5.4)	51 (9.3)	-	-	5 (4.7)	-	7 (12.7)	-	-
FOS	-	183 (33.6)	167 (43.5)	-	-	-	-	-	-
PB	22 (3.3)	-	52 (13.5)	17 (10.4)	-	-	-	-	-
DO	232 (34.8)	223 (41.0)	-	30 (18.4)	-	-	-	-	-
AK	275 (41.2)	387 (71.1)	256 (66.7)	27 (16.5)	35 (33.0)	54 (30.8)	-	19 (39.6)	19 (70.1)
TZP	297 (44.6)	207 (38.0)	223 (58.0)	29 (17.8)	49 (46.2)	23 (13.1)	21 (38.1)	23 (47.9)	23 (85.1)
SAM	-	-	-	63 (38.6)	-	-	-	-	-

Abbreviations: imipenem (IMP); meropenem (MEM); cefazolin (CFZ); cefuroxime (CXM); ceftazidime (CAZ); cefotaxime (CTX); cefepime (FEP); cefoxitin (FOX); ceftaroline (CTP); ampicillin (AMP); amoxicillin-clavulanic acid (AMC); aztreonam (ATM); ciprofloxacin (CIP); trimethoprim-sulfamethoxazole (SXT); tigecycline (TGC); fosfomycin (FOS); polymyxin B (PB); doxycycline; amikacin (AK); piperacillin-tazobactam (TZP); ampicillin-sulbactam.

**Table 3:** Distribution of carbapenem resistance burden among clinical specimens and wards.

	*K. pneumoniae*	*E. coli*	*Pseudomonas* spp.	*Acinetobacter* spp.	*Citrobacter* spp.	*Proteus* spp.	*Morganella* spp.	*Providencia* spp.	*Burkholderia* spp.	*p*-Value
**Clinical specimens *n* (%)**										
Wound	107 (34.6)	51 (22.9)	67 (39.6)	18 (26.9)	16 (35.6)	19 (31.1)	6 (40.0)	7 (36.8)	1 (20.0)	0.00001
Pus	56 (18.1)	43 (19.3)	38 (22.5)	21 (31.3)	13 (28.9)	21 (34.4)	4 (26.7)	8 (42.1)	2 (40.0)	0.461
Urine	20 (6.5)	63 (28.3)	13 (7.7)	1 (1.5)	1 (2.2)	4 (6.6)	1 (6.7)	-	-	0.010
Blood	29 (9.4)	29 (13.0)	17 (10.1)	7 (10.4)	4 (8.9)	9 (14.8)	-	-	2 (40.0)	0.086
Sputum	19 (6.1)	11 (4.9)	5 (3.0)	4 (6.0)	3 (6.7)	2 (3.3)	2 (13.3)	-	-	0.168
Tracheal secretion	24 (7.8)	6 (2.7)	2 (1.2)	5 (7.5)	-	2 (3.3)	-	2 (10.5)	-	0.757
ETT	13 (4.2)	5 (2.2)	6 (3.6)	2 (3.0)	3 (6.7)	-	-	2 (10.5)	-	0.539
Pleural fluid	8 (2.6)	4 (1.8)	3 (1.8)	3 (4.5)	1 (2.2)	2 (3.3)	1 (6.7)	-	-	0.144
Tip cells	9 (2.9)	3 (1.3)	6 (3.6)	1 (1.5)	-	2 (3.3)	-	-	-	0.037
Drain	14 (4.5)	2 (0.9)	5 (3.0)	-	-	-	-	-	-	0.086
CV line	6 (1.9)	3 (1.3)	4 (2.4)	4 (6.0)	2 (4.4)	-	1 (6.7)	-	-	0.136
Tissue	4 (1.3)	3 (1.3)	3 (1.8)	1 (1.5)	2 (4.4)	-	-	-	-	0.00001
Total	309	223	169	67	45	61	15	19	5	
**Clinical wards *n* (%)**										
General surgery	83 (26.9)	57 (25.6)	53 (31.4)	21 (31.3)	13 (28.9)	20 (32.8)	6 (40.0)	8 (42.1)	1 (20.0)	0.548
General medicine	81 (26.2)	35 (15.7)	27 (16.0)	13 (19.4)	7 (15.6)	13 (21.3)	2 (13.3)	2 (10.5)	-	0.0008
ICU	55 (17.8)	41 (18.4)	27 (16.0)	13 (19.4)	5 (11.1)	9 (14.8)	4 (26.7)	2 (10.5)	1 (20.0)	0.069
Dermatology	17 (5.5)	11 (4.9)	16 (9.5)	6 (9.0)	5 (11.1)	4 (6.6)	-	-	-	0.525
Cardiology	19 (6.1)	19 (8.5)	5 (3.0)	2 (3.0)	3 (6.7)	3 (4.9)	-	2 (10.5)	2 (40.0)	0.438
Pediatric medicin	17 (5.5)	7 (3.1)	15 (8.9)	3 (4.5)	5 (11.1)	4 (6.6)	-	2 (10.5)	1 (20.0)	0.838
Nephrology	11 (3.6)	28 (12.6)	7 (4.1)	-	1 (2.2)	3 (4.9)	-	-	-	0.081
Chest medicine	12 (3.9)	11 (4.9)	11 (6.5)	3 (4.5)	1 (2.2)	1 (1.6)	3 (20.0)	-	-	0.491
Orthopedic surgery	11 (3.6)	4 (1.8)	6 (3.6)	4 (6.0)	2 (4.4)	2 (3.3)	-	1 (5.3)	-	0.060
Oncology	1 (0.3)	9 (4.0)	1 (0.6)	2 (3.0)	1 (2.2)	-	-	1 (5.3)	-	0.006
Neurology	2 (0.6)	1 (0.4)	1 (0.6)	-	2 (4.4)	2 (3.3)	-	1 (5.3)	-	0.177
Total	309	223	169	67	45	61	15	19	5	

**Table 4:** Genotypic profile of carbapenem-resistant clinical strains.

Strains	Resistance Profile		Carbapenemase Genes *n* (%)								
	XDR	MDR	*bla*_NDM-1_	*bla*_OXA-48_	*bla*_KPC-2_	*bla*_VIM_	*bla*_IMP_	*bla*_NDM-1_/ *bla*_OXA-48_	*bla*_OXA-48_/ *bla*_VIM_	*bla*_OXA-48_/ *bla*_IMP_	*bla*_VIM_/ *bla*_IMP_
*K. pneumoniae*	27 (11.5)	197 (88.5)	83 (35.5)	69 (29.5)	36 (15.4)	4 (1.7)	-	37 (15.8)	5 (2.1)	-	-
*E. coli*	19 (13.4)	124 (86.6)	68 (47.5)	53 (37.1)	-	3 (2.1)	-	13 (9.1)	6 (4.2)	-	-
*Pseudomonas* spp.	21 (15.7)	112 (84.3)	37 (27.8)	41 (30.8)	-	11 (8.3)	16 (12.0)	16 (12.0)	9 (6.7)	1 (0.7)	2 (1.5)
*Proteus* spp.	3 (6.5)	43 (93.5)	25 (54.3)	17 (39.5)	-	-	1 (2.2)	3 (6.5)	-	-	-
*Acinetobacter* spp.	9 (19.1)	38 (80.9)	29 (61.7)	14 (29.7)	-	1 (2.1)	-	3 (6.4)	-	-	-
*Citrobacter* spp.	2 (7.6)	24 (92.4)	17 (65.4)	9 (34.6)	-	-	-	-	-	-	-
*Providencia* spp.	1 (9.1)	10 (90.9)	5 (45.4)	4 (36.4)	-	-	-	2 (18.2)	-	-	-
*Morganella* spp.	-	6 (100)	2 (33.3)	3 (50.0)	-	-	1 (16.6)	-	-	-	-
*Burkholderia* spp.	-	3 (100)	1 (33.3)	2 (66.7)	-	-	-	-	-	-	-
Total	92	557	267	212	36	19	18	74	20	1	2

**Table 5:** Distribution of carbapenemases among clinical specimens and wards.

	*bla*_NDM-1_	*bla*_OXA-48_	*bla*_KPC-2_	*bla*_VIM_	*bla*_IMP_	*bla*_NDM-1_/ *bla*_OXA-48_	*bla*_OXA-48_/ *bla*_VIM_	*bla*_OXA-48_/ *bla*_IMP_	*bla*_VIM_/ *bla*_IMP_
**Clinical specimens *n* (%)**									
Wound	82 (30.7) *p* = 0.041	78 (36.8) *p* = 0.575	17 (47.2) *p* = 0.123	7 (36.8) *p* = 0.885	-	38 (51.4) *p* = 0.002	6 (30.0) *p* = 0.615	-	1 (50.0) *p* = 0.662
Pus	69 (25.8) *p* = 0.223	49 (23.1) *p* = 0.897	4 (11.1) *p* = 0.072	2 (10.5) *p* = 0.177	9 (50.0) *p* = 0.006	15 (20.3) *p* = 0.496	3 (15.0) *p* = 0.366	-	1 (50.0) *p* = 0.374
Blood	21 (7.9) *p* = 0.221	15 (7.1) *p* = 0.134	6 (16.7) *p* = 0.135	2 (10.5) *p* = 0.883	2 (11.1) *p* = 0.819	13 (17.6) *p* = 0.012	2 (10.0) *p* = 0.944	1 (100)	-
Tracheal secretion	11 (4.1) *p* = 0.965	8 (3.8) *p* = 0.731	-	5 (26.3) *p* < 0.001	2 (11.1) *p* = 0.134	1 (1.4) *p* = 0.198	-	-	-
Sputum	14 (5.2) *p* = 0.641	14 (6.6) *p* = 0.128	-	1 (5.3) *p* = 0.919	-	2 (2.7) *p* = 0.374	-	-	-
Urine	37 13.9) *p* = 0.154	19 (9.0) *p* = 0.129	7 (19.4) *p* = 0.137	-	3 (16.7) *p* = 0.507	1 (1.4) *p* = 0.003	9 (45.0) *p* < 0.001	-	-
Tissue	3 (1.1) *p* = 0.657	2 (0.9) *p* = 0972	-	-	-	1 (1.4) *p* = 0.683	-	-	-
Drain	4 (1.5) *p* = 0.745	4 (1.9) *p* = 0.791	-	-	-	3 (4.1) *p* = 0.094	-	-	-
CV line	3 (1.1) *p* = 0.926	2 (0.9) *p* = 0.816	-	-	2 (11.1) *p* < 0.001	-	-	-	-
ETT	10 (3.7) *p* = 0.301	7 (3.3) *p* = 0.693	2 (5.6) *p* = 0.335	-	-	-	-	-	-
Pleural fluid	8 (3.0) *p* = 0.131	5 (2.4) *p* = 0.652	-	-	-	-	-	-	-
Tip cells	5 (1.9) *p* = 0.415	9 (4.2) *p* = 0.041	-	2 (10.5) *p* = 0.021	-	-	-	-	-
Total	267	212	36	19	18	74	20	1	2
**Clinical wards *n* (%)**									
General surgery	67 (25.1) *p* = 0.170	43 (20.3) *p* = 0.001	16 (44.4) *p* = 0.029	9 (47.4) *p* = 0.064	7 (38.9) *p* = 0.322	36 (48.6) *p* < 0.001	4 (20.0) *p* = 0.392	1 (100)	2 (100)
ICU	74 (27.7) *p* < 0.001	23 (10.8) *p* < 0.001	7 (19.4) *p* = 0.286	2 (10.5) *p* = 0.313	5 (27.8) *p* = 0.373	15 (20.3) *p* = 0.871	1 (5.0) *p* = 0.095	-	-
General medicine	57 (21.3) *p* = 0.971	59 (27.8) *p* = 0.005	6 (16.7) *p* = 0.474	-	1 (5.6) *p* = 0.096	13 (17.6) *p* = 0.391	3 (15.0) *p* = 0.477	-	-
Dermatology	19 (7.1) *p* = 0.321	14 (6.6) *p* = 0.657	4 (11.1) *p* = 0.185	2 (10.5) *p* = 0.401	-	-	-	-	-
Nephrology	11 (4.1) *p* = 0.901	3 (1.4) *p* = 0.020	-	2 (10.5) *p* = 0.141	-	3 (4.1) *p* = 0.982	7 (35.0) *p* < 0.001	-	-
Chest medicine	9 (3.4) *p* = 0.870	9 (4.2) *p* = 0.311	-	-	3 (16.7) *p* = 0.001	-	-	-	-
Cardiology	9 (3.4) *p* = 0.042	*p* = 19 (9.0) *p* = 0.008	-	-	-	5 (6.8) *p* = 0.629	3 (15.0) *p* = 0.060	-	-
Pediatric medicine	9 (3.4) *p* = 0.013	23 (10.8) *p* < 0.001	3 (8.3) *p* = 0.577	1 (5.3) *p* = 0.868	2 (11.1) *p* = 0.376	-	2 (10.0) *p* = 0.468	-	-
Neurology	2 (0.7) *p* = 0.958	3 (1.4) *p* = 0.190	-	-	-	-	-	-	-
Oncology	3 (1.1) *p* = 0.631	3 (1.4) *p* = 0.965	-	3 (15.8) *p* < 0.001	-	-	-	-	-
Orthopedic surgery	7 (2.6) *p* = 0.365	*p* = 13 (6.1) *p* = 0.007	-	-	-	2 (2.7) *p* = 0.728	-	-	-
Total	267	212	36	19	18	74	20	1	2

**Table 6:** Genetic profile of carbapenemase-positive strains.

	Sequence Type (*n*)	Carbapenemases	ESBL Resistance Genes	Replicon Type
*K. pneumoniae*	ST147 (4)	*bla*_KPC-2_	*bla*_SHV_/*bla*_TEM_	IncFII, IncA/C, IncN, IncL/M
	ST147 (9)	*bla*_KPC-2_	*bla*_SHV_/*bla*_CTX-M_	IncFII, IncA/C, IncN, IncL/M
	ST147 (3)	*bla*_KPC-2_	*bla*_SHV_/*bla*_CTX-M_/*bla*_TEM_	IncFII, IncA/C, IncN, IncL/M
	ST147 (13)	*bla*_NDM-1_	*bla*_SHV_/*bla*_CTX-M_	IncFII, IncFIIK, IncA/C, IncN, IncL/M
	ST147 (7)	*bla*_NDM-1_	*bla*_SHV_/*bla*_TEM_	IncFII, IncA/C, IncN, IncL/M
	ST147 (3)	*bla*_NDM-1_	*bla*_SHV_	IncFII, IncA/C, IncN, IncL/M
	ST147 (5)	*bla*_NDM-1_	*bla*_SHV_/*bla*_CTX-M_/*bla*_TEM_	IncFII, IncA/C, IncN, IncL/M
	ST147 (2)	*bla*_NDM-1_	*bla*_CTX-M_	IncFII, IncA/C, IncN, IncL/M
	ST147 (5)	*bla*_NDM-1_	*bla*_CTX-M_/*bla*_TEM_	IncFII, IncA/C, IncN, IncL/M
	ST147 (9)	*bla*_NDM-1_/*bla*_OXA-48_	*bla*_SHV_/*bla*_CTX-M_	IncL/M, IncFII, IncA/C
	ST147 (3)	*bla*_NDM-1_/*bla*_OXA-48_	*bla*_SHV_	IncL/M, IncFII, IncA/C
	ST147 (5)	*bla*_NDM-1_/*bla*_OXA-48_	*bla*_CTX-M_	IncL/M, IncFII, IncA/C
	ST258 (7)	*bla*_KPC-2_	*bla*_SHV_/*bla*_CTX-M_	IncFIIA, IncA/C, IncL/M
	ST258 (3)	*bla*_KPC-2_	*bla*_CTX-M_/*bla*_TEM_	IncFIIA, IncA/C, IncL/M
	ST258 (7)	*bla*_NDM-1_/*bla*_OXA-48_	*bla*_SHV_/*bla*_CTX-M_	IncL/M, IncFII
	ST340 (5)	*bla*_NDM-1_	*bla*_SHV_/*bla*_CTX-M_	IncFII, IncA/C
	ST340 (3)	*bla*_NDM-1_	*bla*_SHV_	IncFII, IncA/C
	ST11 (2)	*bla*_KPC-2_	*bla*_CTX-M_	IncFIIA, IncA/C, IncL/M
	ST11 (5)	*bla*_KPC-2_	*bla*_SHV_/*bla*_CTX-M_	IncFIIA, IncA/C, IncL/M
	ST11 (3)	*bla*_KPC-2_	*bla*_SHV_	IncFIIA, IncA/C, IncL/M
	ST11 (11)	*bla*_NDM-1_	*bla*_CTX-M_	IncFII, IncA/C, IncN, IncL/M
	ST11 (7)	*bla*_NDM-1_	*bla*_SHV_/*bla*_CTX-M_	IncFII, IncA/C, IncN, IncL/M
	ST11 (3)	*bla*_NDM-1_	*bla*_SHV_/*bla*_CTX-M_/*bla*_TEM_	IncFII, IncA/C, IncN, IncL/M
	ST11 (1)	*bla*_NDM-1_	*bla*_SHV_	IncFII, IncA/C, IncN, IncL/M
	ST11 (13)	*bla*_NDM-1_/*bla*_OXA-48_	*bla*_SHV_	IncL/M, IncFII, IncN
	ST14 (7)	*bla*_NDM-1_	*bla*_SHV_/*bla*_CTX-M_	IncFII, IncA/C, IncN, IncL/M, IncFIIK
	ST14 (5)	*bla*_NDM-1_	*bla*_CTX-M_	IncFII, IncA/C
	ST14 (3)	*bla*_NDM-1_	*bla*_SHV_	IncFII, IncA/C
	ST14 (3)	*bla*_NDM-1_	*bla*_TEM_	IncFII, IncA/C
*E. coli*	ST405 (11)	*bla*_NDM-1_	*bla*_CTX-M_	IncFII, IncA/C, IncN, IncL/M
	ST405 (4)	*bla*_NDM-1_	*bla*_SHV_/*bla*_CTX-M_	IncFII, IncA/C, IncN, IncL/M
	ST405 (7)	*bla*_NDM-1_	*bla*_CTX-M_/*bla*_TEM_	IncFII, IncA/C, IncN, IncL/M
	ST405 (3)	*bla*_NDM-1_/*bla*_OXA-48_	*bla*_SHV_/*bla*_CTX-M_/*bla*_TEM_	IncFII, IncL/M
	ST405 (3)	*bla*_NDM-1_/*bla*_OXA-48_	*bla*_SHV_/*bla*_CTX-M_	IncFII, IncL/M
	ST405 (2)	*bla*_NDM-1_/*bla*_OXA-48_	*bla*_CTX-M_/*bla*_TEM_	IncFII, IncL/M
	ST131 (5)	*bla*_NDM-1_	*bla*_SHV_/*bla*_CTX-M_	IncFII, IncA/C, IncN, IncL/M
	ST131 (3)	*bla*_NDM-1_	*bla*_SHV_	IncFII, IncA/C, IncN, IncL/M
	ST131 (1)	*bla*_NDM-1_	*bla*_CTX-M_/*bla*_TEM_	IncFII, IncA/C, IncN, IncL/M
	ST131 (1)	*bla*_NDM-1_/*bla*_OXA-48_	*bla*_SHV_/*bla*_CTX-M_	IncFII, IncL/M
	ST101 (7)	*bla*_NDM-1_	*bla*_SHV_/*bla*_CTX-M_	IncFII, IncN
	ST101 (9)	*bla*_NDM-1_	*bla*_CTX-M_	IncFII, IncN
	ST101 (2)	*bla*_NDM-1_/*bla*_OXA-48_	*bla*_SHV_	IncFII, IncN, IncL/M
	ST69 (7)	*bla*_NDM-1_	*bla*_TEM_	IncFII, IncA/C, IncN, IncL/M
	ST69 (3)	*bla*_NDM-1_	*bla*_SHV_/*bla*_CTX-M_/*bla*_TEM_	IncFII, IncA/C, IncN, IncL/M
	ST69 (1)	*bla*_NDM-1_/*bla*_OXA-48_	*bla*_SHV_	IncFII, IncA/C, IncN, IncL/M
	ST10 (1)	*bla*_NDM-1_/*bla*_OXA-48_	*bla*_SHV_/*bla*_CTX-M_	IncFII, IncN, IncL/M
	ST10 (4)	*bla*_NDM-1_	*bla*_SHV_	IncFII, IncA/C, IncN, IncL/M
	ST10 (7)	*bla*_NDM-1_	*bla*_CTX-M_	IncFII, IncA/C, IncN, IncL/M

## Data Availability

The data used to support the findings of this study are available from the corresponding author upon request.

## Supplementary Materials

The following supporting information can be downloaded at: https://www.mdpi.com/article/10.3390/antibiotics12030525/s1, Table S1: Primer sequences for PCR and sequencing analysis; References [101,102,104,105] belong to Supplementary Materials.
