# Supplementary material for: Estimation, Evaluation and Characterization of Carbapenem Resistance Burden from a Tertiary Care Hospital, Pakistan
Source: Antibiotics (Basel). 2023 Mar 6;12(3):525. doi: 10.3390/antibiotics12030525 (PMC10044297; doi:10.3390/antibiotics12030525)
Supplement: Supplementary file 1 [file antibiotics-12-00525-s001.zip › antibiotics-2253183-supplementary.pdf]

Research Article

Estimation, evaluation and characterization of carbapenem resistance burden from a tertiary care hospital, Pakistan

Aamir Jamal Gondal[1], Nakhshab Choudhry[2], Hina Bukhari[3], Zainab Rizvi[4], Shah Jahan[5] and Nighat Yasmin[1]

[1] Department of Biomedical Sciences, King Edward Medical University, Lahore 54000, Pakistan.
[2] Department of Biochemistry, King Edward Medical University, Lahore 54000, Pakistan.
[3] Department of Pathology, King Edward Medical University, Lahore 54000, Pakistan.
[4] Department of Oral Pathology, de'Montmorency College of Dentistry, Lahore 54000, Pakistan.
[5] Department of Immunology, University of Health Sciences, Lahore 54600, Pakistan.

Correspondence: Dr. Nighat Yasmin, Associate Professor
Postal Address: Department of Biomedical Sciences, King Edward Medical University, Nelagumbad Chowk, Anarkali Bazar, Lahore 54000, Pakistan.
E.mail: drnyasmin@kemu.edu.pk
Cell: +92-321-8566886

Table S1: Primer sequences for PCR and sequencing analysis

	Primer Sequences	Tm °C / Amplicon (bp)
Antimicrobial Resistance Genes **[**14**,** 104**,** 105**]**:		
PCR cycling condition: 40 sec at 95 °C, 30 sec at melting temperature, 30 sec at 72 °C		
*bla*KPC-2	F: gct aca cct agc tcc acc ttc	55 / 989
	R: aca gtg gtt ggt aat cca tgc	
*bla*NDM-1	F: ggg cag tcg ctt cca acg gt	53 / 476
	R: gta gtg ctc agt gtc ggc at	
*bla*NDM-1	F: atg gaa ttg ccc aat att atg c	50 / 813
	R: tca gcg cag ctt gtc ggc cat	
*bla*OXA-48	F: gcg tgg tta agg atg aac ac	52 / 438
	R: cat caa gtt caa ccc aac cg	
*bla*VIM	F: gat ggt gtt tgg tcg cat a	52 / 390
	R: cga atg cgc agc acc ag	
*bla*IMP	F: gga ata gag tgg ctt aay tct c	52 / 232
	R: ggt tta aya aaa caa cca cc	
*bla*SHV	F: ctt tat cgg ccc tca ctc aa	55 / 237
	R: agg tgc tca tca tgg gaa ag	
*bla*TEM	F: cgc cgc ata cac tat tct cag aat ga	55 / 445
	R: acg ctc acc ggc tcc aga ttt at	
*bla*CTX-M	F: atg tgc agy acc agt aar gtk atg gc	55 / 593
	R: tgg gtr aar tar gts acc aga ayc agc gg	
MLST *K. pneumoniae* [101]: 30 sec at 96 °C, 30 sec at melting temperature, 40 sec at 72 °C		
gapA	**F:** tga agt atg act cca ctc acg g	60 / 662
	**R:** aac gcc ttt cat tgc gcc ttc gga a	
infB	**F:** ctc tct gct gga cta cat tcg	52 / 462
	**R:** cgc ttt cag ctc cag aac ttc	
mdh	**F:** ccc aac tgc ctt cag gtt cag	52 / 756
	**R:** cct tcc acg tag gcg cat tcc	
pgi	**F:** gag aaa aac ctg ccg gtg ctg ctg	52 / 566
	**R:** cgg tta atc agg ccg tta gtg gag c	
phoE	**F:** acc tgg cgc aac acc gat ttc ttc	52 / 602
	**R:** ttc agc tgg ttg att ttg taa tcc ac	
rpoB	**F:** ggc gaa atg gcg gaa aac ca	52 / 1075
	**R:** gag tct tcg aag ttg taa cc	
tonB	**F:** ctc tat act tcg gta cat cag gtt	48 / 539
	**R:** cct gtt tgg cgg cca gca cct ggt	
infB2	**F:** act aag gtt gcc tcc ggc gaa gc	60
pgi2	**F:** ctg ctg gcg ctg atc ggc at	60
pgi2	**R:** tta tag cgg tta atc agg ccg t	60
MLST *E. coli* [102]: PCR cycling condition: 30 sec at 95 °C, 40 sec at 55 °C, 40 sec at 72 °C		
dinB	F: gtt ttc cca gtc acg acg ttg tat gag agg tga gca atg cgt a	
	R: ttg tga gcg gat aac aat ttc cgt agc ccc atc gct tcc ag	
icd2	F: gtt ttc cca gtc acg acg ttg taa ttc gct tcc cgg aac att g	
	R: ttg tga gcg gat aac aat ttc atg atc gcg tca cca aay tc	
pabB	F: gtt ttc cca gtc acg acg ttg taa atc caa tat gac ccg cga g	
	R: ttg tga gcg gat aac aat ttc ggt tcc agt tcg tcg ata at	
polB	F: gtt ttc cca gtc acg acg ttg tag gcg gct atg tga tgg att c	
	R: ttg tga gcg gat aac aat ttc ggt tgg cat cag aaa acg gc	
putP	F: gtt ttc cca gtc acg acg ttg tac tgt tta acc cgt gga ttg c	
	R: ttg tga gcg gat aac aat ttc gca tcg gcc tcg gca aag cg	
trpA	F: gtt ttc cca gtc acg acg ttg tag cta cga atc tct gtt tgc c	
	R: ttg tga gcg gat aac aat ttc gct ttc atc ggt tgt aca aa	
trpB	F: gtt ttc cca gtc acg acg ttg tac act ata tgc tgg gca ccg c	

	R: ttg tga gcg gat aac aat ttc cct cgt gct ttc aaa ata tc
uidA	F: gtt ttc cca gtc acg acg ttg tac att acg gca aag tgt ggg tca at
	R: ttg tga gcg gat aac aat ttc cca tca gca cgt tat cga atc ctt
Sequencing	
	oF: gtt ttc cca gtc acg acg ttg ta
	oR: ttg tga gcg gat aac aat ttc
